# Therapeutic dilemmas in young children with lung cancer: a case report and literature review

**DOI:** 10.3389/fonc.2025.1704977

**Published:** 2025-12-09

**Authors:** Qun-xian Zhang, Qiang Guo, Dan Li, Tao Liu, Xiang-Yu Luo, Hua Liu, Jun Zhou, Min Zeng, Jun Zhang, Chen-Yi Lin

**Affiliations:** 1 Department of Cardiothoracic Surgery, Taihe Hospital, Hubei University of Medicine, Shiyan, China; 2 Department of Oncology, Taihe Hospital, Hubei University of Medicine, Shiyan, China

**Keywords:** chest CT scan, GGN, case report, literature review, lung cancer

## Abstract

With the widespread use of plain chest CT scans, the detection of early-stage lung cancer has risen. This manuscript reviews surgical strategies for young patients with early-stage ground-glass nodules (GGNs) indicative of lung adenocarcinoma (LUAD), based on a case study and literature review, aiming to provide clinical insights for management. A 15-year-old adolescent male patient who underwent a routine chest CT, which revealed a ~0.7 cm GGN in the right upper lung. A follow-up CT on April 20, 2025, showed interval growth to approximately 9 × 8 mm in the apical segment. On July 1, 2025, the patient underwent wedge resection of the right upper lung via VATS. Postoperative pathology confirmed minimally invasive LUAD. The patient recovered well and was discharged the following day. A review of literature (2020–2025) identified eight cases of lung cancer in patients ≤18 years. Two patients did not undergo operation due to advanced disease. The remaining underwent surgical resection, with two requiring adjuvant therapy. In summary, lung cancer should be considered in the differential diagnosis of pulmonary nodules detected on plain chest CT. The decreasing age of onset underscores the need for timely intervention to prevent delayed treatment and improve prognosis.

## Background

Lung cancer ranks among the leading causes of cancer incidence and mortality worldwide, comprising non-small cell carcinoma and small cell carcinoma. Surgical management remains the preferred treatment for early-stage disease, offering a favorable prognosis, whereas patients with advanced-stage lung cancer have poor outcomes (1). Many patients are diagnosed late due to the absence of typical symptoms, leading to treatment delays. Therefore, early diagnosis is of critical importance.

**Figure 1:**
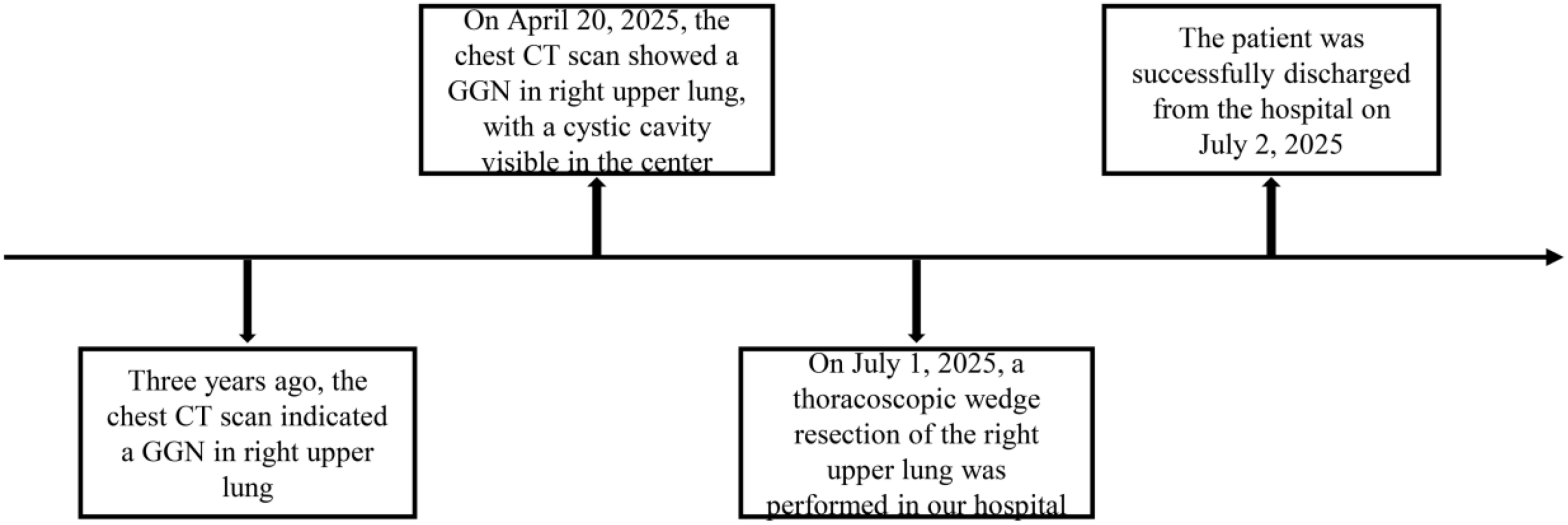
Schematic representation of the follow-up and treatment process of pediatric patients in our hospital.

In recent years, the widespread use of plain chest computed tomography (CT) has significantly increased the detection of lung cancer, particularly following coronavirus disease 2019 (COVID-19) infection, highlighting its role in early diagnosis. However, young children typically undergo plain chest X-rays for routine physical examinations. Lung cancer in this population frequently presents with nonspecific features, leading to delayed or missed diagnoses (2), and potential disease progression. Although the incidence of lung cancer in children is rising, diagnostic strategies, perioperative management, and treatment decision-making remain suboptimal. Therefore, by presenting the treatment course of a young patient (**Figure 1**), supported by a literature review, this study offers new insights and recommendations for the management of pediatric lung cancer.

## Case description

**Figure 2:**
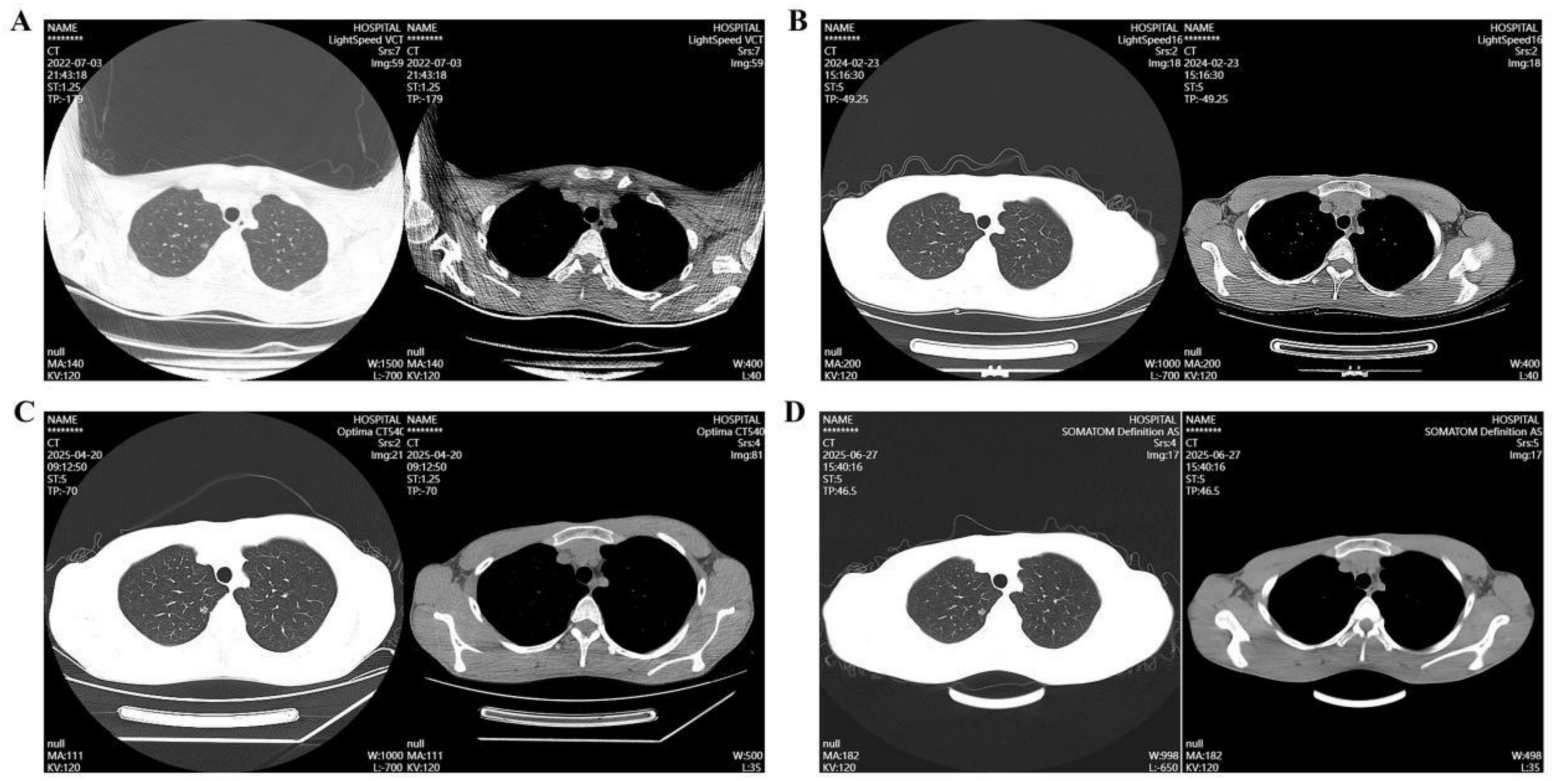
Chest CT scan showing a nodule in the right upper lung of the patient during follow-up (2022-2025). **(A)** July 3, 2022; **(B)** February 3, 2024; **(C)** April 20, 2025; **(D)** June 27, 2025,.

A 15-year-old man presented with a history of long-term, intermittent chest pain. Over three years earlier, chest CT revealed a ground-glass nodule (GGN) in the right upper lung, measuring approximately 0.7 cm in diameter (**Figure 2A**). The patient reported no respiratory symptoms such as cough or expectoration. There was no known family history of genetic disorders or malignancies, and he had no history of smoking or alcohol use. On February 3, 2024, a chest CT performed at our hospital identified a GGN in the right upper lung, approximately the size of a copper coin (**Figure 2B**). Follow-up CT scans on April 20, 2025, and June 27, 2025, revealed a mixed GGN in the apical segment of the right upper lobe with a small cystic cavity. The lesion had enlarged to approximately 9 × 8 mm compared with the previous examination (**Figures 2C, D**).

On admission, physical examination findings were: temperature 36.8°C, respiratory rate 18 breaths/min, pulse 80 beats/min, and blood pressure 129/89 mmHg. The patient was alert, with no palpable lymphadenopathy. Breath sounds were clear bilaterally without rales. Cardiac borders were normal, rhythm was regular, and no pathological murmurs were detected. The abdomen was soft and non-tender, without rebound tenderness or hepatosplenomegaly. No lower-extremity edema was present.

After preoperative evaluation, the patient underwent thoracoscopic wedge resection of the right upper lobe on July 1, 2025. A single-port approach was made through a 2.5 cm incision at the fourth intercostal space along the right midaxillary line. Intraoperative inspection revealed no pleural effusion. The nodule was localized using preoperative non-contrast chest CT. The lesion and surrounding lung tissue were elevated to ensure adequate margins, and the nodule was excised with surrounding parenchyma using a thoracoscopic linear stapler.

**Figure 3:**
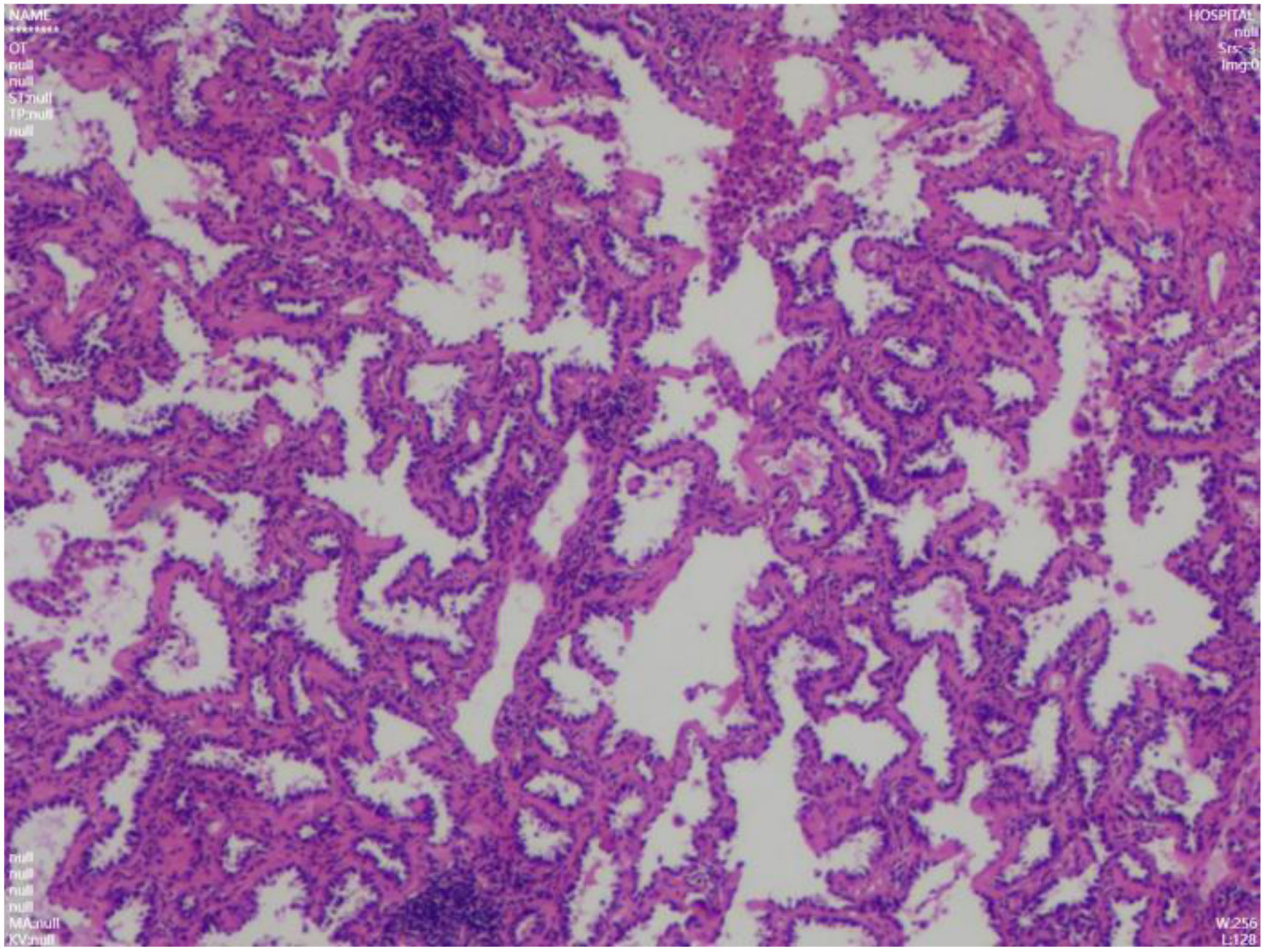
Postoperative pathological examination confirming lung adenocarcinoma.

**Figure 4:**
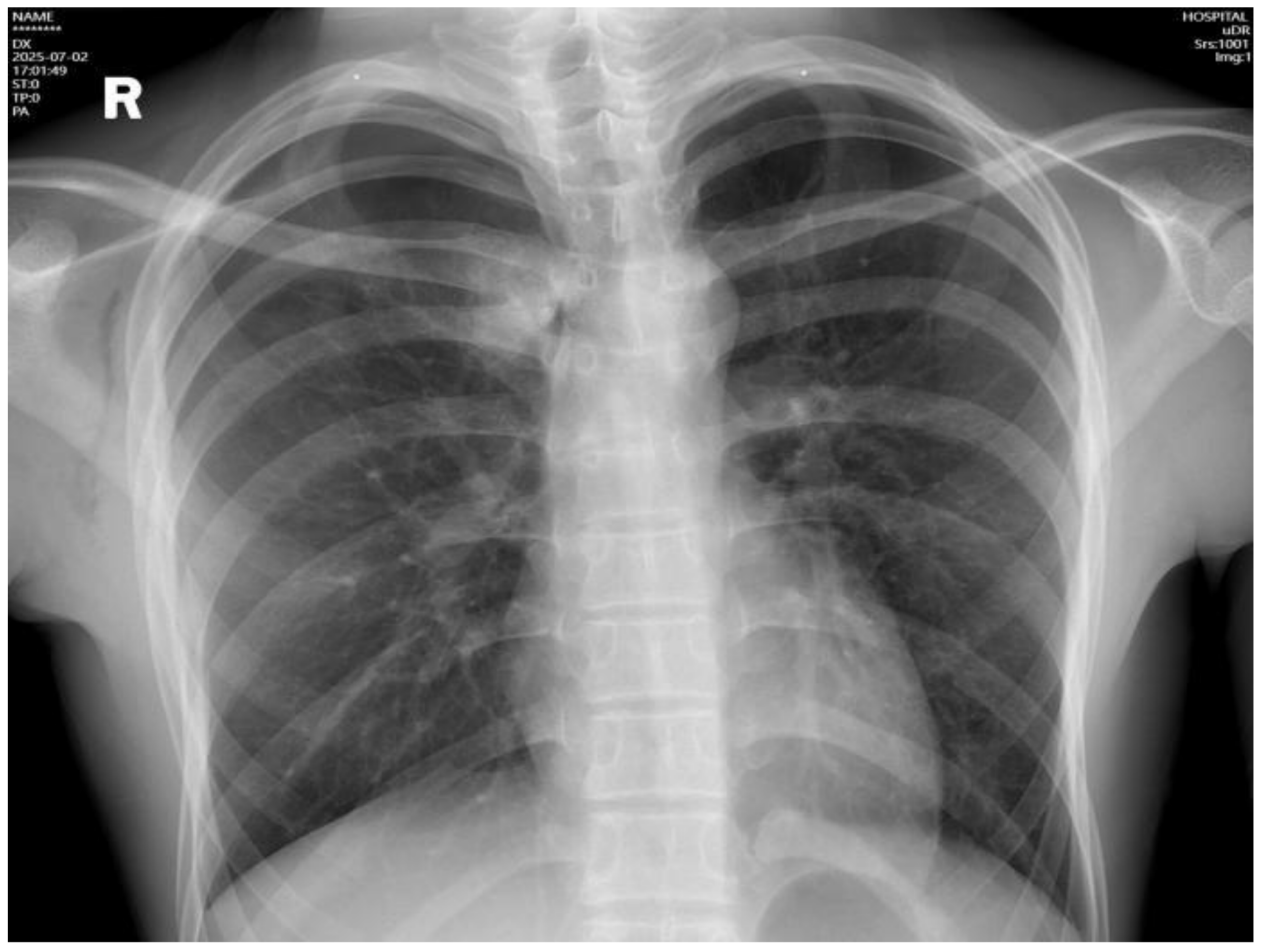
Postoperative chest X-ray demonstrating satisfactory lung recruitment.

Intraoperative frozen section analysis confirmed minimally invasive adenocarcinoma. Hemostasis was meticulously secured. The right lung was re-expanded under direct vision, and the chest tube was removed following complete evacuation of air. Final pathology confirmed minimally invasive lung adenocarcinoma, pT1aN0M0, stage IA1 (**Figure 3**). The tumor measured approximately 0.7 cm in the most significant dimension, with a 0.3 cm invasive component, demonstrating acinar architecture. No pleural involvement, lymphovascular invasion, perineural infiltration, or spread through air spaces was identified. Both surgical and bronchial margins were tumor-free. Lung cancer gene mutation testing was not performed. Postoperative management included symptomatic support with antibiotics (penicillin), mucolytics, and analgesics. The patient’s recovery was uneventful. A chest X-ray on postoperative day 1 showed no significant pneumothorax (**Figure 4**), and the patient was discharged in stable condition. During a phone follow-up, the patient’s family reported that he is doing very well.

## Literature review

A PubMed search of studies published between 2020 and 2025 identified eight case reports of lung GGNs in patients ≤18 years (3–10). The annual distribution included one report in 2020, two each in 2021 and 2022, and one per year from 2023 to 2025. Most cases were reported in female patients (**Table 1**). Three patients were incidentally detected through screening chest CT, and five were found following chest CT performed after symptoms of cough, hemoptysis, or fever. Two patients had comorbidities (diabetes or juvenile systemic sclerosis). None reported smoking or genetic family history. Two patients were diagnosed at advanced stages and received antitumor therapy without surgical resection. Two others were classified as stage IIB postoperatively and underwent adjuvant therapy (**Table 1**).

**Table 1:** Reported cases of LUAD in patients (2020–2025).

Name	Year	Age	Sex	Diseases	Tumor size in CT	Surgical	Stage	Pathology	Ref
Wen et al.	2024	9	Female	–	8×8mm	Yes	IA1	LUAD	(3)
Megaro et al.	2021	17	–	Diabetes	–	No	IV	LUAD	(4)
Zhou et al.	2025	9	Female	–	19×16mm	Yes	IA2	LUAD	(5)
Zhou et al.	2021	13	Male	–	–	No	IV	LUAD	(6)
Wu et al.	2020	17	Female	–	55mm	Yes	IIB	LUAD	(7)
Aliyeva et al.	2022	14	Female	JSS	4mm	Yes	–	Mucous LUAD	(8)
Wang et al.	2022	18	Female	–	58×58×50mm	Yes	IIB	LUAD	(9)
Shahkar et al.	2023	8	Female	–	32×25mm	Yes	IA3	LUAD	(10)

JSS, Juvenile systemic sclerosis.

## Discussion

Plain chest radiography cannot reliably detect or characterize lung GGNs, making chest CT essential for diagnosis. The incidence of GGNs has increased in recent years (11), with more cases reported in younger patients. Greater health awareness has contributed to the broader acceptance of chest CT for detecting pulmonary GGNs. In our case, the lesion was initially detected when the patient was 12 years old during a chest CT scan requested by his parents. The nodule was considered suspicious for early-stage lung cancer. However, given the patient’s asymptomatic status, conservative management was chosen. Over three years of follow-up, the lesion demonstrated slight progression. A repeat CT scan revealed a small cystic cavity within the mixed GGN in the apical segment of the right upper lung, further raising concern for malignancy.

Lung GGNs can frequently be cured with timely intervention. In this case, the nodule was successfully removed via thoracoscopic wedge resection of the right upper lung. Intraoperative frozen section analysis confirmed minimally invasive LUAD, and no additional resection was required. Final staging was pT1aN0M0, stage IA1, consistent with published findings. Among pediatric cases identified in our literature review, the average age was 11.9 years old. Among the eight patients, there were one male, six females, and one with unreported gender. Lung cancer was incidentally detected via screening chest CT in three patients, while the remaining five underwent CT examination after presenting with symptoms such as cough, hemoptysis, or fever. Two patients, diagnosed with advanced disease preoperatively, received neoadjuvant anti-tumor therapy and did not undergo surgical resection. Another two patients were pathologically staged as IIB postoperatively and received adjuvant therapy. This finding underscores the critical importance of early detection in this patient population. Combined analysis of individual case reports and literature reviews suggests that younger age is associated with higher malignancy, reinforcing the value of early surgical treatment.

Diagnosing lung cancer in younger patients remains challenging, as most are asymptomatic with no significant medical history. Few undergo chest CT proactively, leading to missed diagnoses. In this case, the patient underwent a CT for unexplained chest pain, which revealed the GGN—an incidental but fortunate finding. However, whether routine CT screening should be performed in all young patients remains controversial. In addition, this study has some limitations. The postoperative follow-up period for the patient is relatively short, and the patient is currently attending classes at school and unable to come to the hospital for re-examination. This requires us to pay attention to the follow-up results of the patient in the later stage. In conclusion, chest CT is essential for evaluating pulmonary space-occupying lesions. Lung cancer should be included in the differential diagnosis of early-stage findings, particularly as the age of onset continues to decline. Active treatment strategies in young patients are strongly recommended to avoid missing the optimal window for intervention and to improve prognosis.

## Data Availability

The original contributions presented in the study are included in the article/supplementary material. Further inquiries can be directed to the corresponding authors.
